# Unveiling role of sphingosine-1-phosphate receptor 2 as a brake of epithelial stem cell proliferation and a tumor suppressor in colorectal cancer

**DOI:** 10.1186/s13046-020-01740-6

**Published:** 2020-11-23

**Authors:** Luciana Petti, Giulia Rizzo, Federica Rubbino, Sudharshan Elangovan, Piergiuseppe Colombo, Silvia Restelli, Andrea Piontini, Vincenzo Arena, Michele Carvello, Barbara Romano, Tommaso Cavalleri, Achille Anselmo, Federica Ungaro, Silvia D’Alessio, Antonino Spinelli, Sanja Stifter, Fabio Grizzi, Alessandro Sgambato, Silvio Danese, Luigi Laghi, Alberto Malesci, Stefania Vetrano

**Affiliations:** 1 IBD Center, Department of Gastroenterology, Humanitas Clinical and Research Center-IRCCS, Rozzano, Italy; 2 Department of Biomedical Sciences, Humanitas University, Via Rita Levi Montalcini, Pieve Emanuele, Italy; 3 Department of Pathology, Humanitas Clinical, and Research Center-IRCCS, Milan, Italy; 4 Area of Pathology, Department of Woman and Child Health and Public Health, Fondazione Policlinico Universitario A. Gemelli-IRCCS, Rome, Italy; 5 Colon and Rectal Surgery Unit, Humanitas Clinical and Research Center-IRCCS, Rozzano, Italy; 6 Department of Pharmacy, School of Medicine and Surgery, University of Naples Federico II, Naples, Italy; 7 Laboratory of Molecular Gastroenterology, Department of Gastroenterology, Humanitas Clinical and Research Center IRCCS, Rozzano, Italy; 8 Flow Cytometry Core, Humanitas Clinical and Research Center-IRCCS, Rozzano, Italy; 9 Department of Pathology, Faculty of Medicine, University of Rijeka, Rijeka, Croatia; 10 Department of Immunology and Inflammation, Humanitas Clinical and Research Center IRCCS, Rozzano, Italy; 11 Centro di Riferimento Oncologico della Basilicata (IRCCS-CROB), Rionero in Vulture, Italy; 12 Department of Medicine and Surgery, University of Parma, Parma, Italy

**Keywords:** Colorectal cancer, Lgr5, S1PR2, PTEN, Epithelial proliferation

## Abstract

**Background:**

Sphingosine-1-phosphate receptor 2 (*S1PR2*) mediates pleiotropic functions encompassing cell proliferation, survival, and migration, which become collectively de-regulated in cancer. Information on whether *S1PR2* participates in colorectal carcinogenesis/cancer is scanty, and we set out to fill the gap.

**Methods:**

We screened expression changes of S1PR2 in human CRC and matched normal mucosa specimens [*N* = 76]. We compared CRC arising in inflammation-driven and genetically engineered models in wild-type (S1PR2^+/+^) and S1PR2 deficient (S1PR2^−/−^) mice. We reconstituted S1PR2 expression in RKO cells and assessed their growth in xenografts. Functionally, we mimicked the ablation of S1PR2 in normal mucosa by treating S1PR2^+/+^ organoids with JTE013 and characterized intestinal epithelial stem cells isolated from S1PR2^−/−^Lgr5-EGFP- mice.

**Results:**

S1PR2 expression was lost in 33% of CRC; in 55%, it was significantly decreased, only 12% retaining expression comparable to normal mucosa. Both colitis-induced and genetic Apc^+/min^ mouse models of CRC showed a higher incidence in size and number of carcinomas and/or high-grade adenomas, with increased cell proliferation in S1PR2^−/−^ mice compared to S1PR2^+/+^ controls. Loss of S1PR2 impaired mucosal regeneration, ultimately promoting the expansion of intestinal stem cells. Whereas its overexpression attenuated cell cycle progression, it reduced the phosphorylation of AKT and augmented the levels of PTEN.

**Conclusions:**

In normal colonic crypts, S1PR2 gains expression along with intestinal epithelial cells differentiation, but not in intestinal stem cells, and contrasts intestinal tumorigenesis by promoting epithelial differentiation, preventing the expansion of stem cells and braking their malignant transformation. Targeting of S1PR2 may be of therapeutic benefit for CRC expressing high Lgr5.

**Graphical Abstract. Schematic drawing of the role of S1PR2 in normal mucosa and colorectal cancer. In the normal mucosa, S1PR2 is highly expressed by differentiated cells at the upper region of both colon and intestinal crypts (S1PR2 ON), but not by the undifferentiated stem cell at the base of the crypts (S1PR2 OFF), in which acts as a negative proliferative regulator promoting epithelial differentiation. Its loss leads to the expansion of stem cells and reduced levels of PTEN and Axin-2, two negative regulators respectively of PI3K/AKT and Wnt signaling that control β-catenin signaling. The translocation of β-catenin into the nucleus promotes the transcription of target genes involved in the proliferation and malignant transformation. Thereby, S1PR2 works in the intestine as a tumor suppressor:**

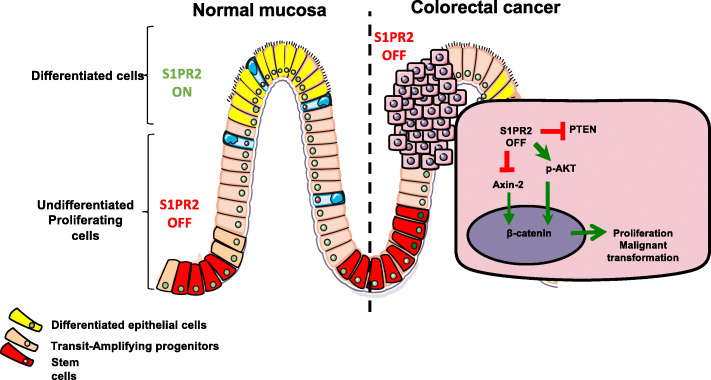

**Supplementary Information:**

The online version contains supplementary material available at 10.1186/s13046-020-01740-6.

## Background

The sphingosine-1-phosphate (S1P) is a pleiotropic and widespread bioactive molecule belonging to the sphingolipid family, a complex group of lipids present in all eukaryotic cells. Previously considered to play only structural functions, sphingolipids are now recognized as key regulators of a myriad of cellular functions in pathophysiological processes [1, 2]. S1P receptors 1–3 (S1PR1, S1PR2, and S1PR3) are expressed ubiquitously, whereas the expression of S1PR4 and S1PR5 is mainly confined to the lymphoid, hematopoietic tissue, and central nervous system [3]. The association with different G proteins activate several downstream pathways contributing to the regulation of many cellular mechanisms [3]. Consequently, considerable interest has been devoted to the S1P/S1PRs axis as potential therapeutic targets for the modulation of several cellular processes. The blockade of S1PR1 is emerging as a new therapeutic approach to control the aberrant leukocyte migration into the intestinal mucosa in inflammatory bowel disease [4]. Conversely, the function of other S1P receptors in the gut has received less attention so far. Recent studies demonstrated the involvement of S1P signaling in several types of cancers [5–9], including colon cancer [10–12]. Colorectal cancer (CRC) is a heterogeneous disease in which different subtypes may be distinguished according to their clinical and molecular features [13]. Intestinal stem cells play an important role in CRC pathogenesis due to their pre-existing proliferative and self-healing behavior, suggesting them to serve as the source for most colorectal cancers [14]. Intestinal epithelial cell proliferation and differentiation are driven by intestinal epithelial stem cells located at the base of crypts, which are either active Leucine-rich repeat-containing G-protein coupled receptor 5 positive (Lgr5+) or quiescent (Lgr5-). According to the traditional ‘bottom-up’ model of CRC development, the transformation of Lgr5+ crypt stem-cells is the principal mechanism initiating the aberrant growth leading to adenomatous polyps, predisposing to cancer [14]. However, the molecular cues driving these events remain to be elucidated.

S1P/S1PR1 axis has been found as a critical key in the link between chronic inflammation and colon cancer by promoting persistent activation of STAT3 in intestinal epithelial cells, which consequently leads to malignant transformation [10]. However, the role of S1P receptors in CRC remains to be determined. mRNA levels of S1PR1, S1PR2, and S1PR3 expression in CRC patients have scarcely been investigated. The only two studies reported controversial results, possibly due to the limited size and heterogeneity of the cohort of patients analyzed. The first study showed a varied expression profile of all three receptors without any trend along CRC tissues analyzed [15], whereas a second one an increased expression of S1PR2 and S1PR3 in tumor samples compared to normal tissue samples [16].

S1PR2 is a critical receptor for the development and progression of different types of cancers and, although its role is controversial depending on the tissue, most data support an anti-tumor function [17]. Indeed, S1PR2 negatively regulates migration and invasion of human melanoma [18], glioblastoma [19], oral squamous cell carcinoma, and gastric cell lines [11] and cell proliferation in human renal tumor cells [20]. Moreover*,* the genetic deletion of S1PR2 promoted the growth of melanoma and Lewis lung carcinomas in vivo [21], supporting S1PR2 as a critical regulator of cell proliferation. A recent study has demonstrated that S1PR2 on intestinal epithelial cells regulates epithelial barrier by preventing CD4 + T-cell proliferation [22]. Nevertheless, its function in epithelial cells remains to be elucidated.

In this study, we demonstrated, for the first time, that S1PR2 functions as a brake on the proliferation of intestinal stem cells. Its inhibition bursts epithelial proliferation and promotes tumor development. In human CRC, the expression of S1P2 is drastically reduced, thus unveiling S1PR2 as a new candidate tumor suppressor gene in colorectal tumorigenesis.

## Materials and methods

### Human tissue collection

Frozen tissue biopsies of 5 adenomas, 39 primary adenocarcinomas with stage II/III pT3-T4, and 16 normal tissues were obtained from fresh tissue biobank collection at Humanitas Clinical and Research Center and proceeded for the mRNA and protein extraction. Healthy tissue was collected at a distance of at least 10 cm from the tumor lesion. No patient had received any therapy before surgery.

The trial set included formalin-fixed, paraffin-embedded tumor specimens from 40 consecutive patients with stage II/III pT3-T4 colorectal adenocarcinoma who underwent surgery and retrieved from the archives of the Department of Pathology of Humanitas Clinical and Research Center-IRCCS from 2011 to 2017. No patients received therapy before surgery (Table 1).

**Table 1:** Clinicopathologic features of colorectal cancer patients (CRC) of the Humanitas Cohort

Cases	40 CRC
**Gender**	
Male	25
Female	15
**Median age** (years, range)	71 (35–81)
**Site**	
Colon right	16 (40%)
Colon left	11 (27,5%)
Rectum	13 (32,5%)
**Histology**	
Mucinous	2 (5%)
Adenocarcinoma	38 (95%)
**TNM staging system**	
**Primary tumor (T)**	
T1	8 (20%)
T2	12 (30%)
T3	11 (27.5%)
T4	9 (22.5%)
**Regional lymph nodes (N)**	
N0	30 (75%)
N1	6 (15%)
N2	4 (10%)
**Distant metastases (M)**	
M0	40 (100%)
M1	0
**Chemotherapy/ neoadjuvant**	
No	40 (100%)
yes	0 (0%)
KRAS *mut*	14 (35%)
KRAS *wilde type*	7 (17,5%)
BRAF *mut*	2 (5%)
BRAF wild type	38 (95%)

The external validation set included tissues of stage II colorectal cancer from Tissue Microarray (TMA) 36 consecutive patients who had undergone surgery from 2006 to 2007 at the Digestive Surgery Clinic, Clinical Hospital Center, and Faculty of Medicine, University of Rijeka, Rijeka, Croatia. Exclusion criteria were age less than 18 or more than 75 years. The study was approved by the Ethics Committee of Humanitas Clinical and Research Center-IRCCS (Rozzano, Italy). All patients gave their written and informed consent.

### Cell culture

All cell lines (SW480, SW620, RKO, HCT116, HT29) derived from human colon cancer were used between 18 and 24 passages, and cultured in Dulbecco’s modified Eagle medium (DMEM; Gibco) supplemented with 10% (v/v) fetal bovine serum, 1 mmol/L l-glutamine, 1 mmol/L sodium pyruvate, 0.1 mmol/L non-essential amino acids, and 100 U/mL) antibiotics (penicillin and streptomycin) at 37 °C in 5% CO2.

### Animals

Male and female C57BL/6 S1PR2^−/−^ mice aged 5–12 weeks old (generated by the team of Dr. Richard L. Proia, NIH, Bethesda, MD) were maintained according to national (D.Lgs 26/2014) and international animal care criteria and used to generate S1PR2^−/−^ and S1PR2^+/+^ littermates in the facility of Humanitas Clinical and Research Center. Female and male C57BL/6 Apc^min/+^ aged 5–12 mice were purchased from the Jackson Laboratory (Stock No: 002020). The double transgenic mouse S1PR2^−/−^ Apc^min/+^ was generated in the facility of the Humanitas Clinical and Research Center by crossbreeding S1PR2^−/−^ and Apc^min/+^ mice. Athymic female CD-1 nude mice (Strain code: 086) were purchased from the Charles River Laboratories. All animals were fed a standard diet and housed in standard polypropylene mouse cages on sawdust bedding. Rooms were kept at 23 °C and maintained on a 12-h light and 12-h dark cycle. All experiments were performed accordingly with the approval from the ethics committees of the Humanitas Clinical and Research Center, in agreement with national (D.Lgs 26/2014) and international animal care criteria. The genotypes were determined by PCR analysis of genomic DNA extracted from tail biopsies by NaOH 60 Mm tail lysis. The primer sequences used for the genotyping of the S1PR2 gene were: 5′- GCA GTG ACA AAA GCT GCC GAA TGC TG-3′, 5′- AGA TGG TGA CCA CGC AGA GCA CGT AG − 3′ and 5′- TGA CCG CTT CTT CGT GCT TTA CGG TAT − 3. The sequences of primers used for the genotyping of the Apc gene were 5′- GCC ATC CCT TCA CGT TAG -3′, 5′- TTC CAC TTT GGC ATA AGG C − 3′ and 5′- GTG CAA TCC ATC TTG TTC AAT − 3′.

### Immunohistochemistry staining

Murine and human samples were fixed in 4% formalin, processed, paraffin-embedded, and sectioned in 2 μm slides. The colonic and intestinal paraffin-embedded tissue slides were deparaffinized, dehydrated with ethanol, and stained with hematoxylin (Dako) and eosin (Diapath) for the single-blinded histological evaluation of the inflammation (Rachmilewitz score) and tumor count or immune-stained. The staining for S1PR2 on murine and human tissue samples was carried out by heating the sections in 10 mM citrate buffer (pH 6.0). The endogenous peroxidase activity was inactivated in hydrogen peroxide 3% for 20 min, and the endogenous mouse IgG and non-specific background were blocked by incubating 30 min in Rodent Block M (Biocare Medical) or Background Sniper (Biocare Medical) respectively murine and human sections. The primary antibody used for the S1PR2 staining was the anti-mouse/human rabbit S1PR2 antibody (Acris;1:200); for 1 h at RT. For the primary antibody detection, the MACH 1 Universal HRP-Polymer (Biocare Medical) and the Betazoid DAB Chromogen Kit (Biocare Medical) have been used. Primary antibodies against Lgr5-GPR49 (Abcam;1:20) were incubated for 2 h at RT in Da Vinci Diluent (Biocare Medical); whereas anti-GFP biotin-conjugated (Invitrogen;1:500), Olfm4 (Cell Signaling; 1:400), Ki67 (Abcam;1:800), Caspase-3 (Cell Signaling; 1:800); CD45 (BD Pharmingen™; 1:100), β Catenin (Abcam; 1:800)1 h at RT. Finally, anti-BrdU (Serotec; 1:600) was incubated 40 min in Da Vinci Green Diluent (Biocare Medical).

### Statistical analysis

Statistical significance was performed using GraphPad Prism 8 (GraphPad software San Diego, CA). The significance of data was evaluated using Paired Student t-tests for comparisons between 2 means. Two-sided nonparametric Mann-Whitney test was used when there was no assumption of normal distribution. One- and Two-way ANOVA tests with Bonferroni’s correction were used to compare the means of 2 or more groups. *P* values below 0.05 were considered to be significant.

## Results

### Reduced to the lost expression of S1PR2 in colorectal cancer

**Fig. 1:**
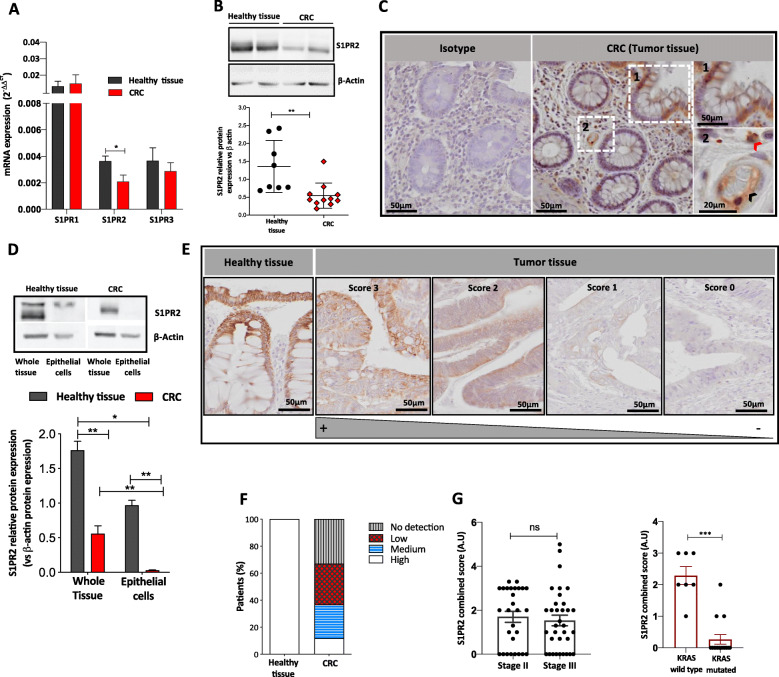
S1PR2 expression in human colorectal cancer. **a** Relative mRNA expression levels of S1PR1, S1PR2 and S1PR3 in CRC (*n* = 39) with stage II/III pT1-T4, and normal colon tissue (*n* = 16) samples. mRNA data are presented as the mean ± SEM and normalized to the expression of human GAPDH and expressed as 2^-ΔΔCt^. The significance was evaluated by two-way ANOVA followed by Bonferroni’s test; *p < 0.05. **b** Representative examples of Western blot analysis (*up panel*) and densitometric analysis (*low panel*) of S1PR2 in human CRC (*n* = 11) and normal colonic mucosa (*n* = 8) samples. Protein levels of S1PR2 were normalized on the β-actin expression. Mean ± SEM, **p = 0.002 by Mann-Whitney test. **c** Representative images of S1PR2 immunostaining of healthy colonic mucosa. In the *left panel* is reported the isotype, whereas in the *right panel* is evidenced the positive staining for S1PR2 in the epithelium, endothelial cells (*black arrow*), and immune cells (*red arrow*). **d** Analysis of S1PR2 in whole tissue and primary epithelial cells isolated from adenocarcinomas and adjacent healthy tissue (*n* = 8). Protein levels of S1PR2 were normalized on the β-actin expression. **e** Immunohistochemical analysis of S1PR2 expression in CRC samples was assessed using a combined score between intensity and extension of the immunoreaction. The images were acquired by the DotSlide system at 20x objective. **f** The stacked bar chart reports the percentage of cases expressing the different intensity of immunostaining in normal (Healthy tissue) and CRC samples. S1PR2 combined score in correlation to (**f**) tumor stage II/III and (**g**) KRAS mutation in CRC. Means ± SEM, *p < 0.05, **p < 0.01, and ***p < 0.001 by one-way ANOVA followed by Bonferroni’s test

To investigate the involvement of S1PR1, S1PR2, and S1PR3 in CRC, we firstly analyzed their mRNA levels in a homogenous cohort of CRC patients (within stages II and III). In the normal mucosa, expression levels of S1PR1 were higher compared to S1PR2 and S1PR3 levels, whereas S1PR2 and S1PR3 expression were comparable to each other/similar (Fig. 1a). Noticeably, in CRC samples, S1PR2 was significantly decreased as compared to normal mucosa (*p* = 0.043), whereas no difference was observed for S1PR1 and S1PR3 levels (Fig. 1a), indicating that only S1PR2 expression is reduced in CRC. Unexpectedly, no difference in the levels of S1P ligand was found between tumor and adjacent healthy tissue (Fig. S1). To address S1PR2 involvement in CRC, we first quantified protein levels, which confirmed the drastic reduction of S1PR2 expression in the tumor (TN0) compared to matched normal mucosa (*p* < 0.01) (Fig. 1b), and second we characterized the tissue distribution of the receptor. Although different cell types, including monocytes and endothelial cells, express S1PR2 in the healthy mucosa (Fig. 1c), we found that S1PR2 was strongly present in the epithelial compartment. Specifically, it was more expressed in differentiated luminal/apical epithelial cells compared to undifferentiated cells residing at the bottom of the crypts (Fig. 1c). The comparison analysis of S1PR2 between the epithelial compartment and the whole tissue confirmed that the receptor is constitutively present on the healthy epithelium and strongly down-regulated on tumor cells (Fig. 1d). To further confirm the above data, we analyzed S1PR2 expression in 40 primary CRC samples and adjacent normal mucosa (Table 1), as well as in a tissue microarray (TMA) cohort consisted of 36 CRC tumors. S1PR2 immunostaining enforced the evidence of its marked reduction in tumor lesions, where it was confined to the epithelial compartment, displaying a heterogeneous modulation varying from a low (intensity score 3) or moderate reduction (intensity score 1–2) to a complete loss (intensity score 0) of the receptor in CRC tissues compared to adjacent normal mucosa (Fig. 1e). Based on a score of immunoreactivity that combines intensity and percentage of S1PR2 immunoreactivity (Supplementary Table 1), 25 (32,89%) out of 76 patients presented no reactivity (score 0); 23 (30,26%) displayed a low (score 1–2); 19 (25%) a medium (score 3–4) and only 9 (11,85%) a high (score 5–6) reaction comparable to healthy tissue (Fig. 1f), with no correlation with CRC stage (*p* = 0.4338) (Fig. 1g). Of relevance, we observed a strong significant reduced immunoreactivity for S1PR2 in CRC samples carrying KRAS mutation (*p* < 0.0001) (Fig. 1g).

### Genetic ablation of S1PR2 increases the susceptibility to develop neoplastic lesions in an in vivo model of colitis-associated colorectal cancer

**Fig. 2:**
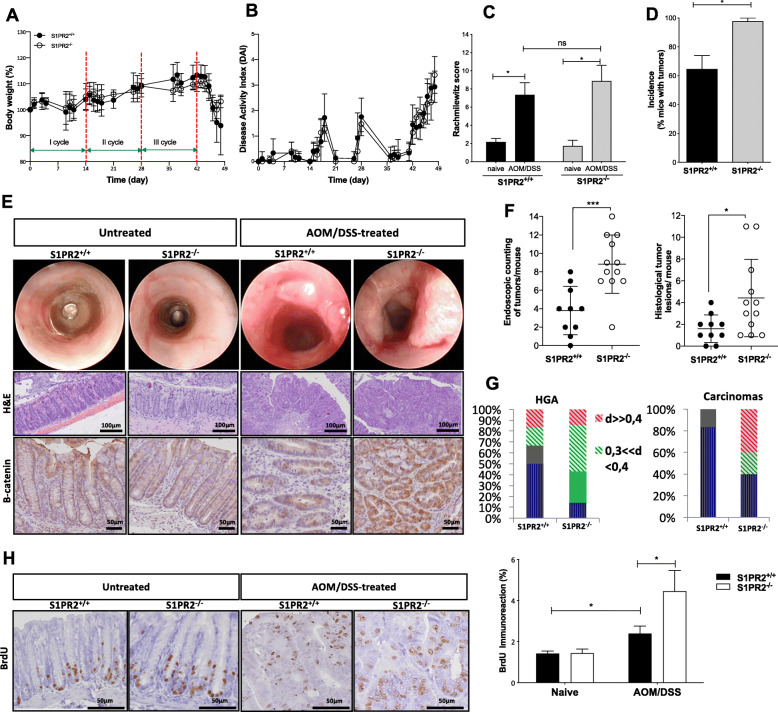
The functional role of S1PR2 in a colitis-associated colon cancer model. Changes in (**a**) body weight, (**b**) disease activity index, and (**c**) histological score of S1PR2^−/−^ (*n* = 12) and S1PR2^+/+^ (*n* = 10) mice after induction of colitis-associated colon cancer by a single AOM injection, followed by three complete oral cycles of 2,5% DSS. **d** Tumor incidence was evaluated macroscopically on day 49. **e** Representative endoscopic (*up panel*) and histological images (*middle panel*) of the intestinal mucosa of S1PR2^−/−^ and S1PR2^+/+^ treated and untreated mice on day 49. Immunohistochemical staining for the expression of β-catenin (*low panel*) was performed on formalin-fixed material. **f** Endoscopic and histological absolute counting of the tumors per mouse. **g** Dimensions (**d**) in millimeters (mm) of high-grade adenoma (HGA) and carcinomas lesions in S1PR2and S1PR2^+/+^ mice. **h** Immunodetection of BrdU in untreated and AOM/DSS treated mice on day 49 and reported as % of nuclear immunoreaction. The images were acquired by the DotSlide system at 20x objective. *p < 0.05; ** *p* < 0.01; and ***p < 0.001 by Mann-Whitney test. Data are representative of 2 experiments

To explore the functional involvement of S1PR2 in CRC development, we took advantage of a mouse model of colitis-associated cancer induced in S1PR2 knockout (S1PR2^−/−^ or KO) mice. S1PR2^−/−^ mice did not show any significant worsening neither in the inflammatory clinical parameters (such as body weight and disease activity index, DAI) nor in the inflammation score compared to S1PR2^+/+^ (Fig. 2a-c). These data were consistent with the results obtained in the acute colitis model, displaying no differences between S1PR2^−/−^ and S1PR2^+/+^ littermates, thus confirming that the loss of S1PR2 does not affect mouse susceptibility to DSS-induced colitis (Fig. S2a-c). In support of this, the analysis of infiltrating CD4-positive T cells combined with the systemic levels of inflammatory cytokines, including IL-6 and IFNγ, did not reveal significant differences between the two groups (Fig. S2e-f). On the other hand, S1PR2^−/−^ mice exhibited a higher tumor incidence than S1PR2^+/+^ mice (100% vs. 63%; *p* = 0.034) Fig. 2d. Furthermore, both endoscopic and microscopic examination revealed that S1PR2-deficient developed a significantly higher number of tumors compared to littermate’s WT animals (*p* < 0.001 and *p* = 0.028, respectively; Fig. 2e *up panel* and Fig. 2f) with increased serum levels of IL-17A, which it has been reported to promote tumor growth (Fig. S2f) [23]. Besides, the histological analysis highlighted a marked increased number of high-grade adenomas (HGA) (1.3 ± 2.109 S1PR2^−/−^ vs. 0.3 ± 0.483 S1PR2^+/+^ mice, *p* < 0.05) with a diameter between 0.3–0.4 mm and an increased number of carcinomas with diameter > 0.4 mm in S1PR2^−/−^ compared to S1PR2^+/+^ mice (2.5 ± 3.6 S1PR2^−/−^ vs. 1 ± 1.2 S1PR2^+/+^mice, *p* < 0.05) (Fig. 2e *middle panels* and Fig. 2g). The difference in the number of low-grade adenomas (LGA) between the two groups did not reach significance (0.3 ± 0.8 S1PR2^−/−^ vs. 0.3 ± 0.6 S1PR2^+/+^ mice, *p* = 0.1) (Fig. S2d). Altogether, these data point to an enhanced cancer susceptibility, coupled to a faster/higher tumor growth rate in the S1PR2^−/−^ background under inflammatory conditions. Interestingly, the immunostaining for nuclear ß-catenin was strongly positive in S1PR2 deficient mice compared to littermate WT mice (Fig. 2e), *low panels*). We next assessed whether the higher incidence of tumors in S1PR2^−/−^ was also associated with a deregulated cell growth by intraperitoneally injecting BrdU 24 h before sacrifice. No difference was observed in healthy conditions between naive and tumor-bearing mice. In contrast, S1PR2^−/−^ tumor-bearing mice displayed an increased cell proliferation in tumor lesions compared to tumor-bearing S1PR2^+/+^ ones (Fig. 2h). The immunoexpression of anti-caspase 3 showed lower apoptotic cells in the tumor region compared to healthy surrounding regions of both S1PR2^+/+^ and S1PR2^−/−^ mice, indicating an enhanced survival of cancer cells (Fig. S3). However, the comparable survival rate observed between the two groups excludes a function of S1PR2 in controlling cell survival (Fig. S3).

### Loss of S1PR2 following *APC* mutation in colon carcinogenesis

**Fig. 3:**
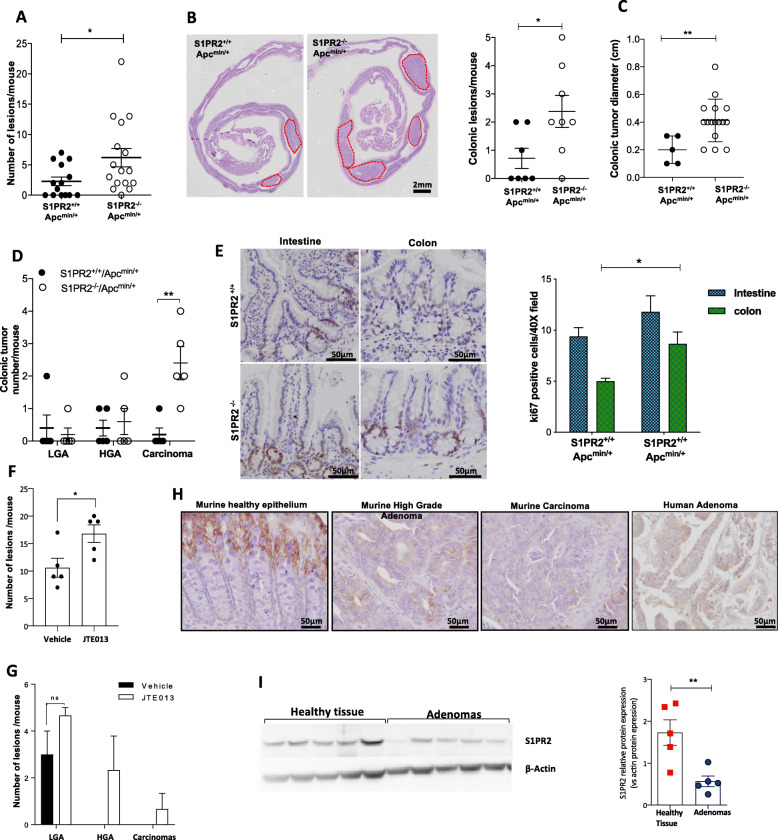
Loss of S1PR2 an early event in the intestinal tumorigenesis. **a** Tumor load in S1PR2^−/−^/Apc^min/+^ and S1PR2^+/+^/Apc^min/+^ mice at 21 weeks of age. **b** Macroscopic examination and quantification of colonic tumors in mice (*red dotted line* show the tumors). **c** Size and **d** histological classification as low (LGA) and high (HGA)-grade adenomas and carcinomas of colonic lesions. **e** Immunodetection of Ki67 in the small intestine and colon of mice. The percentage of positive Ki67 cells for crypt was counted in 40 fields of view. Mean ± SD, *n* = 5; *p < 0.05; ** *p* < 0.01 by unpaired parametric t-test. **f-g** Graphs are reporting the number and histological classification of tumors in Apc^min/+^ mice after 5 weeks of oral administration of JTE013 or vehicle. Mean ± SD, *n* = 4, *p* = 0.049 by unpaired parametric t-test. **h** Immunostaining of S1PR2 in S1PR2^+/+^/Apc^min/+^ mice and in human intestinal adenomas. The images were acquired by the DotSlide system at 20x objective. **i** Western blot analysis (*left panel*) and densitometric analysis (*right panel*) of S1PR2 in human adenomas (*n* = 5) and normal colonic mucosa (*n* = 5) samples. Protein levels were normalized on the β-actin expression. Significance was evaluated by the Mann-Witney test *p < 0.05 and **p < 0.01

To further gain deeper insight into the role of S1PR2 in intestinal tumorigenesis, we also exploited an Apc^Min/+^ mouse model that spontaneously develop multiple polyps in the small intestine [24]. S1PR2 deficiency in Apc^min/+^ at 21 weeks old led to a significant increase of total tumor load over the entire gastrointestinal tract compared with their littermates S1PR2^+/+^Apc^min/+^ mice (*p* < 0.05) (Fig. 3a), which was well evident in the distal colon *(p* = 0.033) (Fig. 3b-c). In keeping with the observation in the DSS mouse model, we also observed a significant increase in the size of tumors in S1PR2^−/−^Apc^min/+^ compared to S1PR2^+/+^Apc^min/+^ mice (*p* = 0.005) (Fig. 3c). In parallel, histologic examination highlighted a significantly higher number of carcinomas in S1PR2^−/−^Apc^min/+^ (2.40 ± 0.51) compared to S1PR2^+/+^Apc^min/+^ mice *p* = 0.041 (Fig. 3d). No difference was observed in LGA and HGA lesions number between the two groups (Fig. 3d). Accordingly, the Ki67 immunostaining showed a significantly increased cell proliferation in the colon of S1PR2^−/−^Apc^min/+^ compared to S1PR2^+/+^Apc^min/+^ mice (Fig. 3e) (*p* = 0.02). We found no difference in the small intestine tumor burden between the two groups (Fig. 3e). To address the role of S1PR2 during the early phases of tumor development, we inhibited pharmacologically S1PR2 in Apc^Min/+^ mice at 10 weeks of age, before the appearance of both intestinal and colonic tumors, by the specific S1PR2 inhibitor (JTE013). JTE103 inhibitor accelerated tumor formation in Apc^min/+^ mice in comparison to the vehicle (Fig. 3f) along the gastrointestinal tract. Moreover, while vehicle-treated Apc^min/+^ mice developed only low-grade adenomas, the Apc^min/+^ mice treated with the JTE013 inhibitor broadened high-grade adenomas and carcinomas (Fig. 3g), corroborating the loss of S1PR2 as an accelerator of tumor development and de-differentiation. S1PR2 immune-histochemical analysis in intestinal tissue of Apc^min/+^ mice revealed a strong decrease of the receptor in the epithelial compartment of both high-grade adenomas and carcinomas compared to the normal epithelium (Fig. 3h).

### Loss of S1PR2 is an early event in the pathogenesis of colorectal cancer

To validate this feature in humans, we analyzed S1PR2 expression in 5 human tubulovillous adenomas with moderate focal dysplasia, which is considered as an early precancerous lesion. All adenoma samples showed a significant reduction of S1PR2 compared to healthy mucosa (Fig. 3h-i), thus corroborating the hypothesis that the loss of S1PR2 in the epithelial compartment plays a key role in colorectal tumorigenesis and that it likely occurs in the early phase of cancer development.

### The overexpression of S1PR2 reduces the tumorigenicity of human CRC-derived epithelial cells in vivo

**Fig. 4:**
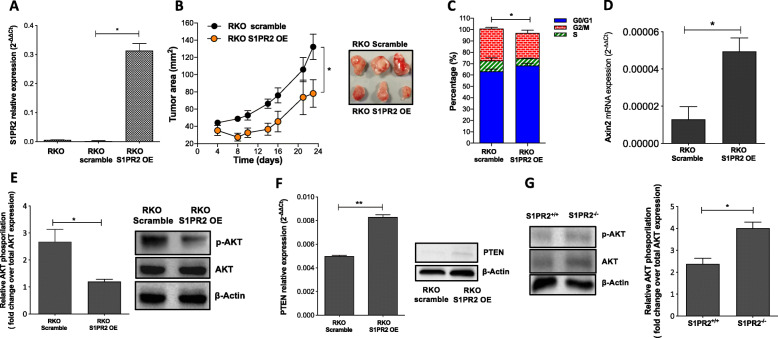
Cellular processes and pathways impacted by S1PR2. **a** Relative mRNA levels of S1PR2 in the RKO cancer cell line before and after overexpression (OE) of S1PR2. **b** In vivo tumor growth of S1PR2-overexpressing GFP-RKO (3 × 10^6^) vs. scramble cells over 23 days after injection. **c** Cell cycle analysis on recovered RKO-derived tumors. **d** Relative mRNA levels of Axin2 in RKO cells. **e** Western blot analysis (*left panel*) and densitometry (*right panel*) analysis of total (AKT) and phosphorylated AKT (p-AKT) in RKO cells. **f** mRNA (*right panel*) and protein (*right panel*) levels of PTEN. **g** Western blot (*left panel*) and densitometry (*right panel*) analysis of phosphorylated AKT (p-AKT) in the mucosa of AOM/DSS treated S1PR2^−/−^ and S1PR2^+/+^ mice. mRNA is mean ± SEM, normalized to the expression of GAPDH and expressed as 2^-ΔCt^. The significance was evaluated by the Mann-Witney test, whereas the cell cycle by one-way ANOVA followed by Bonferroni’s test; *p < 0.05; ** *p* < 0.01. Data are representative of 2 independent experiments; *n* = 4

To gain insight into the mechanisms by which the loss of S1PR2 promotes tumorigenesis, we explored the role of S1PR2 as a brake of tumor proliferation and a potential tumor suppressor gene in vivo. To this end, we firstly examined the endogenous expression of S1PR2 in four metastatic colon cancer cells SW620, RKO, HCT116, and HT29. In line with our previous data, S1PR2 expression was deficient in all cancer cells (Fig. S4a). Then, to test whether S1PR2 acts as a brake of tumor proliferation, we used lentivirus-mediated overexpression of S1PR2 in RKO (RKO-S1PR2-OE), which exhibited, at least in our hands, the highest infection efficiency among all cell lines negative for S1PR2. Overexpression efficiency was verified by RT-PCR (Fig. 4a). The effect of S1PR2 overexpression on the in vivo tumor cell growth was measured over 23 days after subcutaneous injection of RKO cells in female CD-1 nude mice. S1PR2 overexpression attenuated tumor growth with statistical significance (*p* < 0.05) compared to the scramble (Fig. 4b). Cell cycle analysis on the recovered tumors by flow cytometry demonstrated that most RKO cells overexpressing S1PR2 arrested at the G0/G1 phase, while a substantially higher fraction of cells from scramble tumors were in S and G2/M phases compared to S1PR2 overexpressing ones (*p* < 0.05) (Fig. 4c). Based on in vivo experiments in which the loss of S1PR2 promoted the pathological accumulation of nuclear ß-catenin that, in turn, can control cell cycle, we examined whether S1PR2 exerted a direct role in this event. Interestingly, in contrast to scramble cells, RKO-S1PR2-OE displayed significantly higher levels of Axis inhibition protein 2 (*Axin2*) gene (Fig. 4d) that enhances the formation of the beta-catenin destruction complex and therefore prevents the nuclear translocation of ß-catenin.

S1PR2 has been shown to inhibit cell migration in cancer cell lines [19, 20]. To address whether S1PR2-overexpression affected migratory and invasive properties of metastatic RKO cells, we performed transwell migration and invasion assays and analyzed the expression of some genes that support invasive capacities. S1PR2- overexpression did not affect the in vitro migratory capacity of RKO cells (Fig. S4b) neither in the mRNA expression levels of matrix metalloproteinases such as MMP1 and 2 (Fig. S4c), both genes involved in the distant metastasis development in CRC [25]. In line with these features, no difference was found between RKO scramble and RKO S1PR2 OE cells in distant organs such as the liver, mesenteric lymph nodes, adipose tissue, and colon (Fig. S4d) of mouse xenograft model. Overall, these findings supported the key role of S1PR2 in arresting tumor growth and excluded its potential function in controlling the migratory capacity of epithelial tumor cells. Recently S1PR2 has been involved in the growth of hepatocellular carcinoma cells through the activation of PI3K/AKT signaling [26]. To investigate whether, in CRC cells, the modulation of S1PR2 can also drive the activation of the PI3K/AKT pathway, which is highly expressed in the RKO cell line [27], we quantified the protein levels of the phosphorylated AKT in RKO scramble and RKO S1PR2 OE cells. The overexpression of S1PR2 significantly reduced the phosphorylation of AKT (Fig. 4e). It augmented both mRNA and protein levels of Phosphatase and tensin homolog deleted on chromosome ten (PTEN), a negative regulator of the PI3K/AKT pathway (Fig. 4f). These results point out S1PR2 as a regulator of PTEN. To validate this hypothesis in an in vivo tumor model, we quantified pAKT in S1PR2^+/+^ and S1PR1^−/−^ tumor-bearing mice. As expected, the loss of S1PR2 augmented AKT levels and its phosphorylation in the mucosa of KO mice (Fig. 4g).

### S1PR2 inhibition impacts intestinal stem cell expansion

The observation that the S1PR2 receptor is mainly expressed at the top of the intestinal crypts, while its expression is lower at the bottom where to reside intestinal stem cells, may support a differential expression of S1PR2 between differentiated intestinal epithelial and stem cells.

**Fig. 5:**
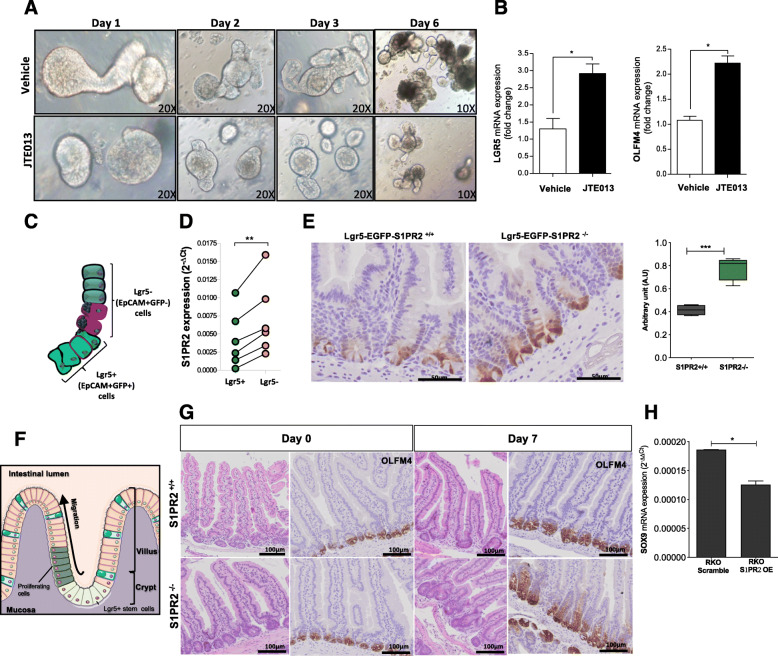
Deletion of S1PR2 promotes the expansion of intestinal stem cells. **a** Organoid development in the presence of JTE013 (10 μM) or vehicle over 6 days. Images were acquired by an inverted light microscope at 10 and 20x objectives. **b** mRNA analysis of LGR5 and OFLM4 in organoids on day 6. **c** Schematic distribution of epithelial progenitor stem cells Lgr5+ (EPCAM+GFP+) and differentiated epithelial cells Lgr5- (EPCAM+GFP-) in the crypts of Lgr5-EGFP-IRES-creERT2 mice. **d** S1PR2 mRNA expression in sorted EPCAM+ GFP positive (Lgr5+) and EPCAM+ GFP negative (Lgr5-) isolated from Lgr5-EGFP-IRES-creERT2 mice (*n* = 6). Data as a mean ± SEM of 6 independent experiments. ** *p* = 0.003 by a paired t-test. **e** Immunostaining of LGR5 and its quantification on intestinal sections of Lgr5-EGFP-S1PR2^−/−^ and Lgr5-EGFP-S1PR2^+/+^ mice. **f** Schematic overview of intestinal epithelial regeneration showing expansion and differentiation of Lgr5 stem cells that move upward into the villus allowing a rapid regeneration of the epithelium. **g** Impaired expression of OLFM4 in the small intestine of S1PR2^−/−^ and S1PR2^+/+^ mice at day 0 and 7 of X-ray irradiation. Data are mean ± SEM and representative of 3 experiments (*n* = 4). **h** mRNA levels of SOX9 in RKO-OE and scramble cells, presented as mean ± SEM, normalized to the expression of GAPDH and expressed as 2^-ΔΔCt^. *p < 0.05; ** *p* < 0.01; and ***p < 0.001 by Mann-Witney test

To verify whether S1PR2 is functionally involved in the proliferation and differentiation of intestinal stem cells, organoids isolated from naive WT mice were maintained in vitro for 6 days in the absence or presence of JTE013. Typically, intestinal organoids cultures tend to exhibit extensive budding of crypt-like domains (Fig. 5a *up panels*). In the presence of JTE013, organoids appeared with more cyst-like morphology characterized by a small number of uncomplete branches (Fig. 5a *low panels*). The round-shape of organoids raised the suspicion that JTE013 could prevent the differentiation of epithelial cells. Indeed, the levels of Olfm4 and Lgr5 stemness markers were significantly higher after JTE013 compared to untreated organoids (Fig. 5b), proving that the loss of S1PR2 maintains the organoids in an undifferentiated status. To gain deep insight into this aspect, we analyzed the expression of S1PR2 in stem (EpCAM+Lgr5-GFP+) and differentiated (EpCAM+Lgr5-GFP-) intestinal epithelial cells isolated from Lgr5-EGFP-IRES-creERT2 mice (Fig. 5c). Accordingly, EpCAM+Lgr5-GFP+ stem cells expressed lower levels of S1PR2 compared to differentiated cells (EpCAM+Lgr5-GFP-) (Fig. 5d). In addition, Lgr5 + GFP immunostaining confirmed an increased number of Lgr5 positive cells in S1PR2^−/−^ mice compared to their littermates Lgr5-EGFP-S1PR2^+/+^ (Fig. 5e).

### Loss of S1PR2 impairs mucosal regeneration in vivo

To assess whether the deregulation of intestinal stem cell proliferation and differentiation participates in the impairment of mucosal regeneration in S1PR2 deficient mice in vivo, which may contribute to intestinal tumorigenesis, we analyzed the regeneration of mucosal structure in S1PR2^−/−^ and S1PR2^+/+^ mice after irradiation. Based on previous studies showing the time of mucosal regeneration following the irradiation [28], the destruction of the normal crypt-villus axis starts after 2 days in association with the expansion of undifferentiated Lgr5+ cells for replacing proliferating cells that, within 6–7 days, renew the intestinal mucosa structure by migrating from the bottom to the top along the crypt-villus axis (Fig. 5f). Both S1PR2^−/−^ and S1PR2^+/+^ mice displayed substantial bodyweight loss within 7 days after irradiation, reflecting the damage to the intestinal mucosa (Fig. S4). After this time, both groups of mice started to gain their body weight in support of the recovery of mucosal damage. Although no difference was observed in the bodyweight recovery between S1PR2^+/+^ and S1PR2^−/−^ mice (Fig. S4), the intestinal mucosa structure presented differently (Fig. 5g). Despite the presence of signs of a regenerative process still in progress, the mucosa of S1PR2^+/+^ mice recovered intestinal damage. It restored intestinal integrity by normalizing the villus height and crypt depth. Differently, the mucosa of S1PR2^−/−^ mice, while showing a recovery of villi height, displayed elongated and enlarged crypts characterized by an increased number of undifferentiated cells, as evidenced by the strong immune-positivity for Oflm4 (Fig. 5g). To further corroborate the key role of S1PR2 in arresting the expansion of intestinal stem cells, we analyzed in RKO-S1PR2-OE and scramble cells the expression of transcription factor Sex-determining region Y (SRY)-box 9 (SOX9), which is linked to stem cell maintenance [29] and implicated in CRC. The overexpression of S1PR2 reduced significantly SOX9 levels compared to RKO scramble cells (Fig. 5h), supporting S1PR2 as a brake for the expansion of intestinal stem cells.

## Discussion

The relevance of the S1P pathway in the development of the CRC has emerged in the last years [10, 12]. However, the overall function of the S1PRs in colorectal tumorigenesis is still controversial [15, 16] and not entirely understood. Our current observations revealed: i) a not significant modulation of S1P levels in CRC patients; ii) higher expression of S1PR1 among the receptors in the normal mucosa; iii) a marked decrease only of S1PR2 in primary CRC already at an early phase (adenoma) of tumor development. Accordingly, S1PR2 deficient mice displayed increased susceptibility to experimental models of CRC. Although various cell types can express S1PR2 in the healthy mucosa, the modulation of the receptor in CRC was confined to the epithelium compartment. A recent study has demonstrated that S1PR2 on intestinal epithelial cells regulates epithelial barrier by preventing CD4 + T-cell proliferation [22]. Nevertheless, its function in epithelial cells is scanty. We showed for the first time that S1PR2 is strongly expressed by differentiated epithelial cells at the upper region of both colon and intestinal crypts, but not at the base by undifferentiated stem cells, which are essential for the regeneration of the epithelium layer. The precise mechanisms that finely control the survival, proliferation and self-renewal of stem cells remain largely unknown. However, perturbations to this delicate balance leading to an excessive self-renewal would expand the stem cell pool at the base of the crypt, increasing the risk of intestinal tumorigenesis [30, 31]. Specifically, targeted ablation of the Lgr5 stem cell population in cancerous tissues revealed that it is dispensable for primary tumor maintenance [32, 33]. Here, we demonstrated by in vitro and in vivo studies that the loss of S1PR2 promotes the expansion of Lgr5-expressing stem/progenitor cells in the gut. Its overexpression in RKO cells resulted in the reduction of SOX9, a marker linked to stem cell maintenance [29], and in an accumulation of cells in the G0/G1 phase and a concomitant reduction of cells in the S and M phases, supporting S1PR2 as a master switch of intestinal self-renewal and differentiation of intestinal stem cells. Wnt/β-catenin signaling works as the primary driving force behind these processes in the intestinal crypts [34]. Lgr5 is a marker of intestinal stem cells with a well-defined function in the promotion of Wnt/β-catenin signalling [35] via modulation of the expression of adenomatous polyposis coli (APC) protein and β-catenin [36].

Consistent with these data, in addition to increased Lgr5-expressing stem cells, S1PR2^−/−^ mice showed a higher nuclear β-catenin accumulation compared to wild type mice. In the canonical Wnt cascade, β-catenin is the key effector responsible for the transduction of the signal to the nucleus. It is maintained into the cytoplasm at low levels and targeted for ubiquitination and degradation by a “destruction complex” that contains APC protein [24]. By crossing mice carrying a mutation in Apc and S1PR2 deficient mice, we observed acceleration of tumor formation, corroborating the hypothesis that the cross-talk of S1PR2 with intracellular Wnt/β-catenin signals can critically regulate proliferative responses and tumor development. Interestingly, Axin2, a negative regulator of the Wnt/β-catenin signaling pathway, which promotes the phosphorylation and degradation of β-catenin [37], was significantly upregulated in the cells overexpressing S1PR2 compared to scramble cells. It is reasonable to hypothesize that targeting Axin 2 S1PR2 regulates the degradation of β-catenin, thus preventing the transcription of genes involved in CRC development. Several studies supported the anti-tumor function of the S1PR2 acting as a negative regulator of the proliferation and migration of cancer cells [11, 19, 20, 38]. However, the modulation of S1PR2 did not impact the migratory properties of RKO cancer cells, thus indicating that the migration capacities of these cells are S1PR2-independent. S1PR2 may intersect Wnt signals alternatively by modulation of Phosphatidylinositide-3-kinase (PI3K) [39], which activates β-catenin mediated transcription through AKT phosphorylation [40]. Enhanced PI3K signaling, either by mutations or through the loss of expression of its antagonist PTEN, can be observed in a variety of human malignancies, including colon cancer [41, 42]. Accordingly, AKT is constitutively activated in the RKO cell line due to the inactivation of PTEN. Of note, AKT phosphorylation was significantly down-regulated after overexpression of S1PR2, which correlated with increased levels of PTEN, indicating a key role of S1PR2 in negatively controlling PI3K-AKT pathways. How relevant is S1P ligand or whether other ligands participate in the activation of PTEN in RKO cells need to be better addressed. The cellular stress observed in the RKO cells overexpressing S1PR2 when cultured in a complete deprivation of S1P allows us to speculate a crucial role of S1P ligand in the activities of these cells (*data not shown*). Consistent with in vitro finding, the mucosa of AOM/DSS S1PR2^−/−^ mice showed higher levels of phosphorylated AKT compared to that from S1PR2^+/+^ mice.

## Conclusions

Based on our observations, S1PR2 protein may contrast the intestinal tumorigenesis by promoting epithelial differentiation, preventing the expansion of stem cells, and blocking their malignant transformation through the suppression of β-catenin nuclear translocation and β-catenin transcriptional activity. Therefore, tumor cells gain a massive advantage by its loss of function. Nevertheless, naïve S1PR2^−/−^ mice showed normal architecture of the gut without developing tumor formations within their lifetime. It is reasonable that the absence of spontaneous tumorigenesis in S1PR2 deficient mice could be due to possible compensatory effects involving other pathways in the normal state that fail upon inflammation or in association with additional mutations.

Particularly, S1PR2 expression negatively correlated with CRC mutated for KRAS, which is associated with poor patient prognosis in CRC and with the resistance to the therapy [43]. This finding raises S1PR2 as a useful marker for early identification of this molecular subgroup of CRC. How S1PR2 expression is linked to this subgroup needs to be clarified. Recently, it has been reported that CRC mutated for KRAS express different miRNA profiles [44], including miR-130a [45], which modulates S1PR2 expression. Indeed, the inhibition of miR-130a-3p by specific inhibitor augmented S1PR2 levels [46]. It is likely that in the initiation process of CRC, normal epithelial cells acquire oncogenic mutations through the interaction between internal and external factors that lead to the downregulation of S1PR2, which in turn triggers an excessive epithelial self-renewal and tumor development. Therefore, targeting of S1PR2 may be of therapeutic benefit for CRC expressing high Lgr5 and of a prevention strategy for CRC development.

## Supplementary Information

**Additional file 1: Supplementary Figure 1.** S1P levels in human colorectal cancer. Quantification of Sphingosine 1 phosphate (S1P) in adenocarcinomas and healthy colon tissue by spectrometry assay (*n* = 7). The levels of S1P are reported as pmol/mg. Data are presented as the mean ± SEM, and significance was evaluated by performing a non-parametric test.

**Additional file 2: Supplementary Figure 2.** Clinical parameters and inflammation score in S1PR2^−/−^ and S1PR2^+/+^ mice after DSS-induced colitis. Acute colitis was induced in S1PR2^−/−^ and S1PR2^+/+^ mice by adding filtered 3% DSS to drinking water for 9 days. Representative graphs showing (A) the changes in body weight and (B) disease activity index (DAI) during the entire experiment. (C) The inflammatory status of the colonic mucosa was evaluated accordingly to the Rachmilewitz score by histological analysis. Data are presented as the mean ± SEM, and significance was evaluated by the Mann-Whitney test. Data is representative of 3 experiments (*n* = 5). (D) Representative histological images of CD45 cells of the colonic mucosa by immunohistochemistry of S1PR2^−/−^ and S1PR2^+/+^ mice after AOM/DSS-induced colitis-associated cancer. The images were acquired by the DotSlide system at 20x objective. The quantification of CD45 is reported as the CD45 immunoreactive area/40X field. (E) Serum levels of IL-6, IFNy, and IL-17A in S1PR2^−/−^ and S1PR2^+/+^ mice after AOM/DSS-induced colitis-associated cancer. Data are presented as the mean ± SEM, and significance was evaluated by the Mann-Whitney test. Data is representative of 2 experiments (*n* = 6). (F) Low-grade adenoma (LGA) in S1PR2^−/−^ and S1PR2^+/+^ mice after AOM/DSS induced colitis. S1PR2^−/−^ (*n* = 12) and S1PR2^+/+^ (*n* = 10) mice. Means ± SEM.

**Additional file 3: Supplementary Figure 3.** Analysis of survival rate of the colonic mucosa of S1PR2^−/−^ and S1PR2^+/+^ mice after AOM/DSS induced colitis-associated cancer. The tissues (healthy and tumor regions) were evaluated by immunohistochemical analysis with an anti-cleaved caspase-3 antibody. The images were acquired by the DotSlide system at 20x objective. Apoptotic cells (*blue arrows*) were examined microscopically at 40X magnification. Data are presented as the mean ± SEM, and significance was evaluated by the Mann-Whitney test. Data is representative of 2 experiments (*n* = 6).

**Additional file 4: Supplementary Figure 4.** Gene expression analysis in colon-rectal cancer cell lines and in S1PR2-overexpressing RKO cells. (A) S1PR2 mRNA expression levels in the indicated CRC cell lines. (B) Representative images of migratory and invasive cells (magnification, 4x) are shown. Cell migration and invasiveness were reported as the number of cells in 40 fields of view. (C) Relative expression of metalloproteinases 1–2 (MMP1–2) in S1PR2-overexpressing RKO and scramble cells by qRT-PCR (2 ^-∆∆Ct^ method). Data are shown as means ± SEM and are representative of 2 independent experiments, each constituted by three replicates. (D) Ex vivo macroscopic examination of organs (liver, adipose tissue, mesenteric lymph nodes (MLN) and xenograft tumor) in nude mice injected with S1PR2-overexpressing and scramble cells by an imaging system (IVIS) using GFP labeled cells. Data are representative of 2 independent experiments; *n* = 4.

**Additional file 5: Supplemental Figure 5.** Bodyweight changes in S1PR2^−/−^ and S1PR2^+/+^ mice after 7 days of X-ray irradiation at 9 Gy. Data are presented as the mean ± SEM, and significance was evaluated by the Mann-Whitney test. Each group of data is representative of 3 experiments (*n* = 4).

**Additional file 6.** Corresponds to supplementary information related to the material and methods.

## Data Availability

The datasets used and/or analyzed during the current study are available from the corresponding author on reasonable request.

## Abbreviations

S1PR: Sphingosine-1-Phosphate Receptor
CRC: Colorectal Cancer
LGR5: Leucine-rich repeat-containing G-protein coupled receptor 5
EGFP: Enhanced Green Fluorescent Protein
APC: Adenomatous polyposis coli
Min: Multiple intestinal neoplasia
PTEN: Phosphatase and tensin homolog deleted on chromosome ten
S1P: Sphingosine-1-phosphate
STAT3: Signal transducer and activator of transcription 3
TMA: Tissue Microarray
KO: Knockout
DAI: Disease activity index
DSS: Dextran Sulfate Sodium
WT: Wild Type
HGA: High-grade adenomas
LGA: Low-grade adenomas
BrdU: Bromodeoxyuridine
OE: Overexpression
Axin2: Axis inhibition protein 2
MMP: Matrix metalloproteinase
PI3K: Phosphatidylinositide-3-kinase
EpCAM: Epithelial cell adhesion molecule
IRES: Internal ribosome entry site
ERT2: Estrogen receptor 2
Oflm4: Olfactomedin 4
SRY: Sex-determining region Y
SOX9: Sex-determining region Y (SRY)-box 9
AOM: Azoxymethane
miRNA: microRNA
RT-PCR: Reverse transcriptase-polymerase chain reaction
p-AKT: Phosphorylated AKT
GAPDH: Glyceraldehyde 3-phosphate dehydrogenase
