# Supplementary figures and images for: Unveiling role of sphingosine-1-phosphate receptor 2 as a brake of epithelial stem cell proliferation and a tumor suppressor in colorectal cancer
Source: J Exp Clin Cancer Res. 2020 Nov 23;39:253. doi: 10.1186/s13046-020-01740-6 (PMC7682101; doi:10.1186/s13046-020-01740-6)

Supplementary Figure 1

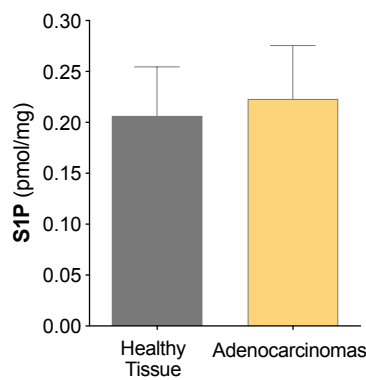

Supplement: Supplementary file 1 — Additional file 1: Supplementary Figure 1. S1P levels in human colorectal cancer. Quantification of Sphingosine 1 phosphate (S1P) in adenocarcinomas and healthy colon tissue by spectrometry assay (n = 7). The levels of S1P are reported as pmol/mg. Data are presented as the mean ± SEM, and significance was evaluated by performing a non-parametric test. [file 13046_2020_1740_MOESM1_ESM.pdf]

Supplementary Figure 2

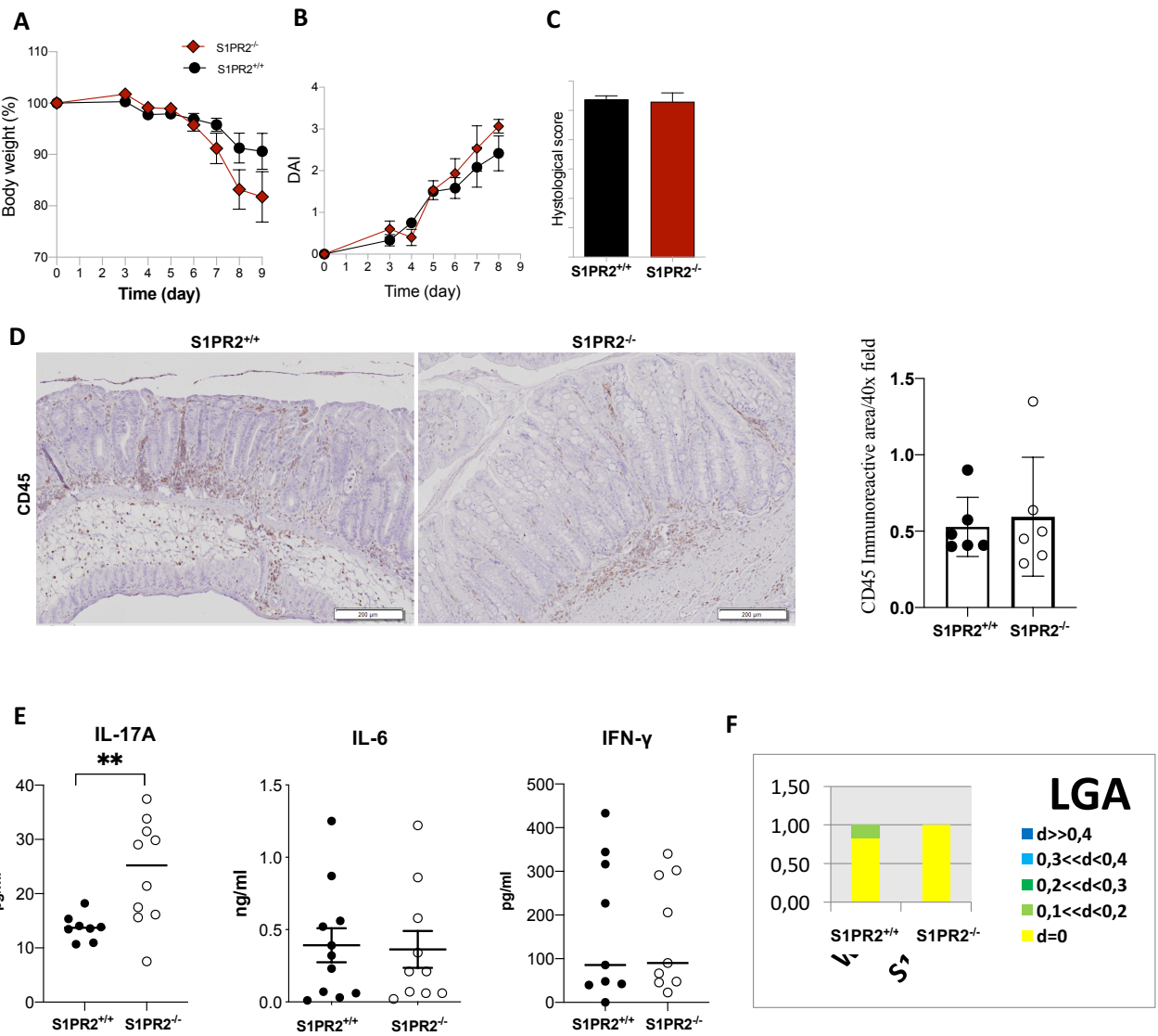

Supplement: Supplementary file 2 — Additional file 2: Supplementary Figure 2. Clinical parameters and inflammation score in S1PR2−/− and S1PR2+/+ mice after DSS-induced colitis. Acute colitis was induced in S1PR2−/− and S1PR2+/+ mice by adding filtered 3% DSS to drinking water for 9 days. Representative graphs showing (A) the changes in body weight and (B) disease activity index (DAI) during the entire experiment. (C) The inflammatory status of the colonic mucosa was evaluated accordingly to the Rachmilewitz score by histological analysis. Data are presented as the mean ± SEM, and significance was evaluated by the Mann-Whitney test. Data is representative of 3 experiments (n = 5). (D) Representative histological images of CD45 cells of the colonic mucosa by immunohistochemistry of S1PR2−/− and S1PR2+/+ mice after AOM/DSS-induced colitis-associated cancer. The images were acquired by the DotSlide system at 20x objective. The quantification of CD45 is reported as the CD45 immunoreactive area/40X field. (E) Serum levels of IL-6, IFNy, and IL-17A in S1PR2−/− and S1PR2+/+ mice after AOM/DSS-induced colitis-associated cancer. Data are presented as the mean ± SEM, and significance was evaluated by the Mann-Whitney test. Data is representative of 2 experiments (n = 6). (F) Low-grade adenoma (LGA) in S1PR2−/− and S1PR2+/+ mice after AOM/DSS induced colitis. S1PR2−/− (n = 12) and S1PR2+/+ (n = 10) mice. Means ± SEM. [file 13046_2020_1740_MOESM2_ESM.pdf]

Supplementary Figure 3

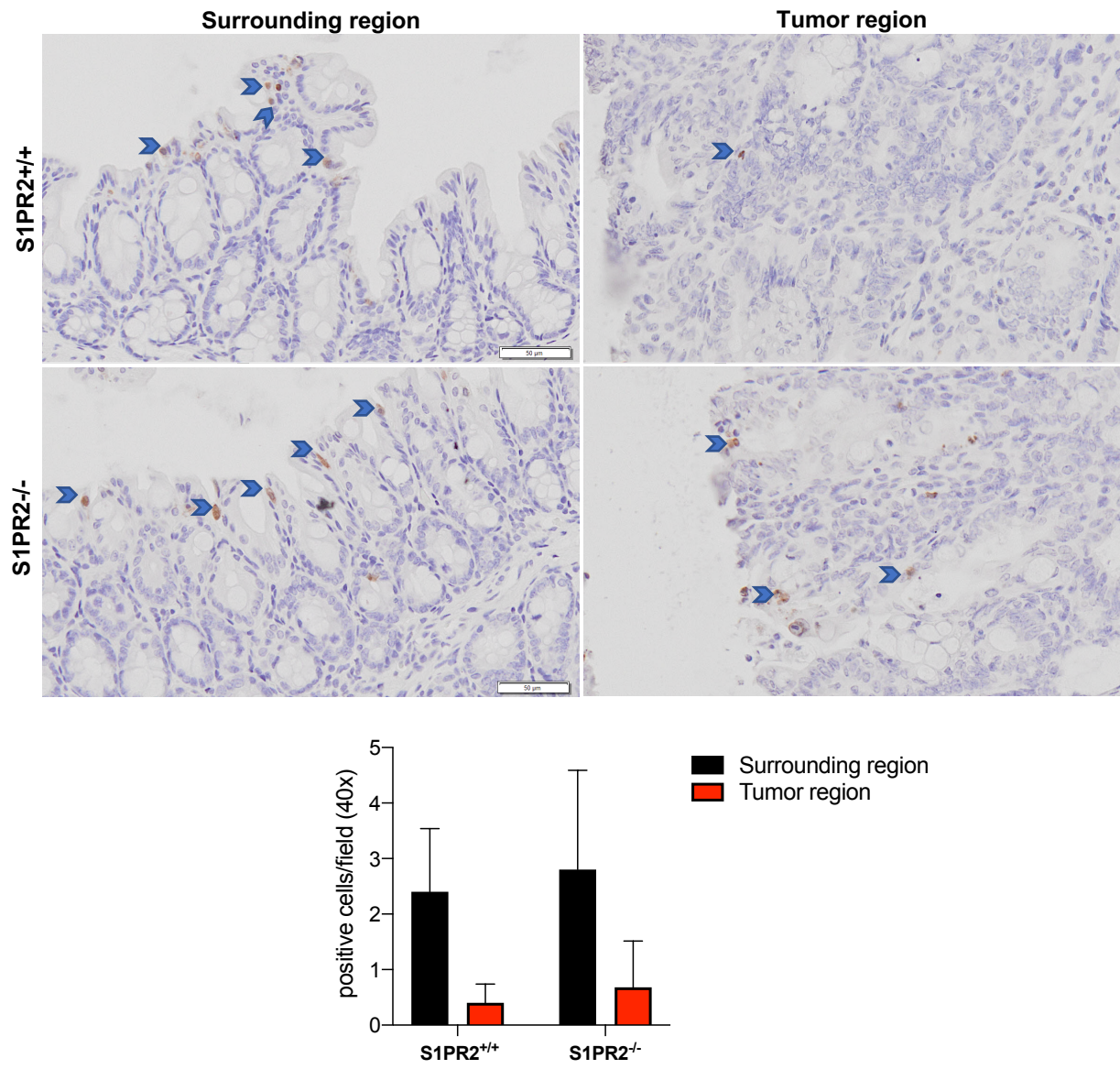

Supplement: Supplementary file 3 — Additional file 3: Supplementary Figure 3. Analysis of survival rate of the colonic mucosa of S1PR2−/− and S1PR2+/+ mice after AOM/DSS induced colitis-associated cancer. The tissues (healthy and tumor regions) were evaluated by immunohistochemical analysis with an anti-cleaved caspase-3 antibody. The images were acquired by the DotSlide system at 20x objective. Apoptotic cells (blue arrows) were examined microscopically at 40X magnification. Data are presented as the mean ± SEM, and significance was evaluated by the Mann-Whitney test. Data is representative of 2 experiments (n = 6). [file 13046_2020_1740_MOESM3_ESM.pdf]

Supplementary Figure 4

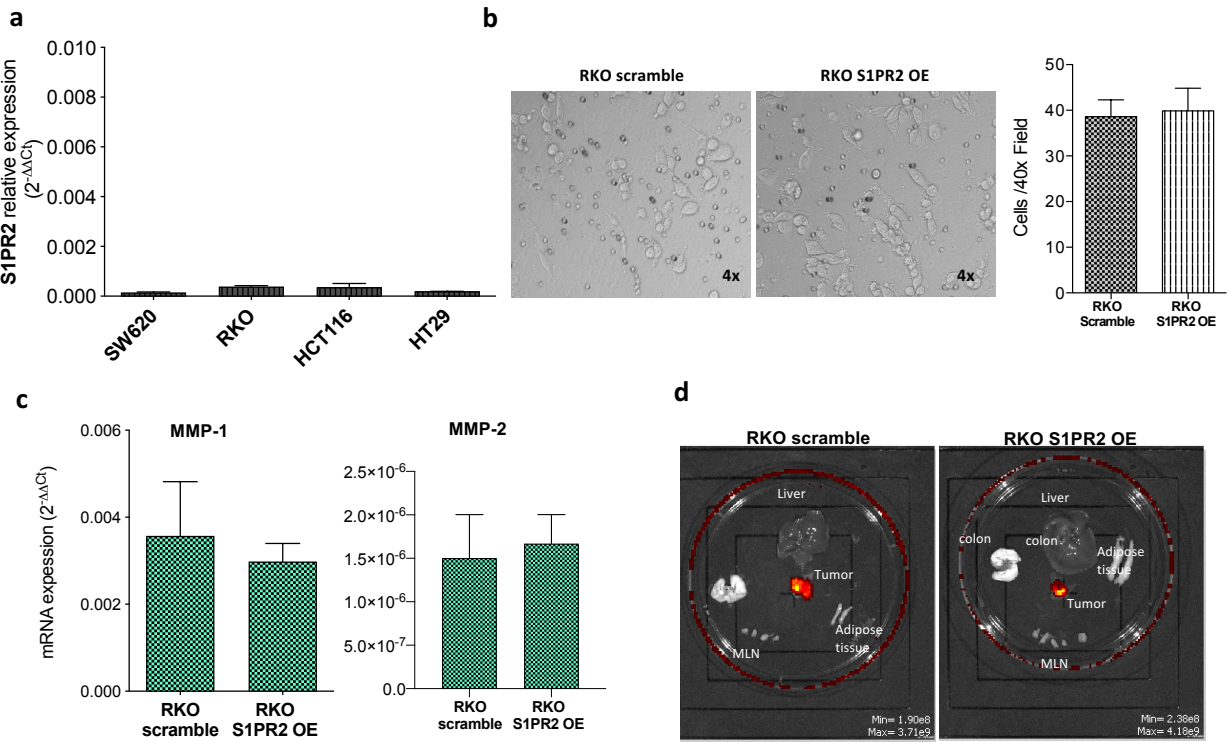

Supplement: Supplementary file 4 — Additional file 4: Supplementary Figure 4. Gene expression analysis in colon-rectal cancer cell lines and in S1PR2-overexpressing RKO cells. (A) S1PR2 mRNA expression levels in the indicated CRC cell lines. (B) Representative images of migratory and invasive cells (magnification, 4x) are shown. Cell migration and invasiveness were reported as the number of cells in 40 fields of view. (C) Relative expression of metalloproteinases 1–2 (MMP1–2) in S1PR2-overexpressing RKO and scramble cells by qRT-PCR (2 -∆∆Ct method). Data are shown as means ± SEM and are representative of 2 independent experiments, each constituted by three replicates. (D) Ex vivo macroscopic examination of organs (liver, adipose tissue, mesenteric lymph nodes (MLN) and xenograft tumor) in nude mice injected with S1PR2-overexpressing and scramble cells by an imaging system (IVIS) using GFP labeled cells. Data are representative of 2 independent experiments; n = 4. [file 13046_2020_1740_MOESM4_ESM.pdf]

Supplementary Figure 5

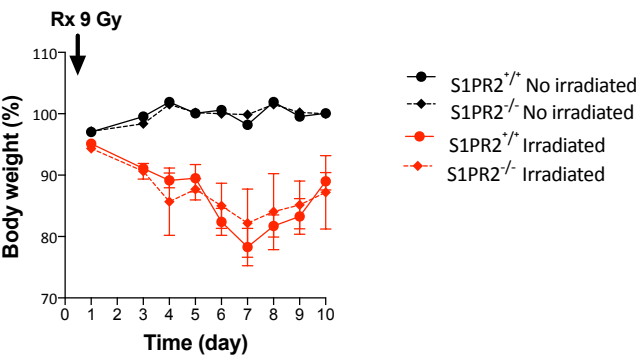

Supplement: Supplementary file 5 — Additional file 5: Supplemental Figure 5. Bodyweight changes in S1PR2−/− and S1PR2+/+ mice after 7 days of X-ray irradiation at 9 Gy. Data are presented as the mean ± SEM, and significance was evaluated by the Mann-Whitney test. Each group of data is representative of 3 experiments (n = 4). [file 13046_2020_1740_MOESM5_ESM.pdf]
