# Supplementary material for: Unveiling role of sphingosine-1-phosphate receptor 2 as a brake of epithelial stem cell proliferation and a tumor suppressor in colorectal cancer
Source: J Exp Clin Cancer Res. 2020 Nov 23;39:253. doi: 10.1186/s13046-020-01740-6 (PMC7682101; doi:10.1186/s13046-020-01740-6)
Supplement: Supplementary file 6 — Additional file 6. Corresponds to supplementary information related to the material and methods. [file 13046_2020_1740_MOESM6_ESM.docx]

**SUPPLEMENTARY INFORMATION**

**Immunoreactive Scoring system.**

The final score of immunoreaction was calculated as described by Klein et al^1^ (**Supplemetary** **Table 1**). Briefly, the percentage of labelled was assessed as 0 = no labeled cells; 1 = < 30%; 2= 30-60%; and 3= > 60%. The intensity was calculated from 0 to 3, where 0 was no reaction; 1 weak, 2 mild, and 3 strong intensity.

**Supplementary Table 1. Immunoreaction scoring system**

| A % of IHC + labeled cells | B intensity of IHC reaction | Final score (A + B) |
| --- | --- | --- |
| 0 = 0 | 0 = no reaction | No reaction = 0 |
| 1 = <30 | 1 = weak | Low = 2 |
| 2 = 30-60 | 2 = mild | Medium = 3-4 |
| 3 = >60 | 3 = strong | High = 5-6 |

**Intestinal Epithelial Cell isolation**

*Human Intestinal Epithelial Cells*. Mucosal samples were first incubated in 2 Mm DTT at 37°C for 15 minutes to remove the residual mucus and then digested mechanically into smaller pieces and incubated in a chelating compound ethylenediaminetetraacetic acid (EDTA) 1Mm at 37°C for 30 minutes. After this time, the mucosal pieces resuspended in a solution supplemented with 2.5% fetal calf serum (FCS) (Sigma-Aldrich) Hepes (Sigma-Aldrich) 20mM and Penicillin/Streptomycin (Sigma-Aldrich) 100 units/ml were agitated for 1-2 min on the stirrer at maximum speed. Whole crypts were collected from following through with a 100-µm cell strainer, and their viability was determined with a 0,4% Trypan blue solution. Final centrifugation at 1300rpm for 5 minutes at 4° C allowed the disgregation of the crypts; single epithelial cells were subsequently counted, pellet, and finally stored at -80°C.

*Murine intestinal stem cells*. Lgr5-expressing stem cells were isolated from Lgr5-EGFP-IRES-creERT2 heterozygous mice aged from 6 to 8 weeks, that express EGFP protein under the control of Lgr5 promoter following the procedure described by Mahe et al^2^. The intestines were extracted from the abdominal cavity, washed to remove the feces and opened lengthwise. The tissue was cut in small pieces and incubated in Phosphate-Buffered Saline (PBS) 2mM EDTA at 37°C for 30 minutes. Then, the crypts were collected by shaking the tissue and filtering with a 70-µm cell strainer. They were further dissociated in single cells after 90 minutes of incubating in TryPLE Express supplemented with 10uM Y27632 (Invitrogen, Carlsbad, CA, USA) and filtering with a 40um cell strainer. To specifically isolate epithelial cells, the single-cell suspension was stained with the anti-mouse CD326 (EpCAM) eFluor 450 Monoclonal Antibody, clone G8.8 (eBioscience) and sorted by FACS. Dead cells were stained by using a LIVE/DEAD cell viability assay kit (Invitrogen, Carlsbad, CA, USA). Live EpCAM+Lgr5-GFP+ and EpCAM+Lgr5-GFP- cells were sorted into RNAlysis buffer provided by the RNAeasy micro kit (QIAGEN) supplemented with 1% β-mercaptoethanol to directly lysate the cells for the mRNA extraction.

**Matrigel invasion assay**

Scrumble and overexpressed S1PR2 RKO cells (3x10^4^ cells per well) were allowed after serum starvation to migrate across the precoated inserts (Corning Matrigel Invasion Chamber, BioCoat) at 37°C for 24 hours. Then unmigrated cells from the top of the chamber were removed, and the migrated cells were viewed using an inverted microscope with x10 objective (Olympus IX51).

**Proliferation Assay**

The cell cycle was evaluated by the use of the BD Pharmigen ^TM^ 647 EdU Click Proliferation Kit (cat.n°: 565456). According to the protocol, 1x10^6^ cells were co-stained with a DNA dye 7-AAD, and cell populations were segmented by flow cytometry into the G0/G1-phases (2N DNA content, EdU-negative), S-phase (2N-4N DNA content, EdU-positive), or G2/M-phases (4N DNA content, EdU-negative).

**Animal experiments**

The acute colitis model was induced in C57BL/6 S1PR2 ^-/-^ (KO) and S1PR2 ^+/+^ (WT) mice by administration of dextran sodium sulfate (DSS) (MP Biomedicals) 3% *ad libitum* in their drinking water for nine days. The inflammation-driven colon carcinogenesis model was induced by a single intraperitoneal injection with the Azoxymethane (AOM Sigma) 10 mg per kg body weight, followed by four cycles of DSS 2.5% in drinking water *ad libitum* for four days spaced out by ten days. The mice were monitored during the entire experiment for body weight changes, bleeding, and consistency of stool three times for a week. The disease activity index (DAI) was evaluated according to the criteria as reported previously^3^. The number of tumors was recorded by endoscopy (Coloview system, STORZ) under general anesthesia with 100 mg/kg intraperitoneal ketamine and 10mg/kg xylazine, at the last DSS cycle and by histology on fixed colon and small intestine paraffin-embedded. Histological analysis of inflammatory status was performed by a single-blinded pathologist accordingly to Rachmilewitz score^3^. S1PR2^-/-^ Apc^min/+^ and S1PR2^+/+^Apc^min/+^ littermates have been sacrificed at 14 or 21-weeks old, and the colon and intestine tissues were collected.

S1PR2^+/+^Apc^min/+^mice received every two other day oral gavage intake of a specific S1PR2 inhibitor, JTE013 (10009458, Cayman Chemical) 10 mg/kg, or vehicle for five weeks after weaning. C57BL/6 S1PR2 ^+/+^ and S1PR2 ^-/-^ mice were irradiated with X-ray at 9 Gy (RADGIL X-ray generator) and monitored for body weight changes daily for seven days. On the day of sacrifice, the intestines were recovered and fixed by 10% formaldehyde for histological examination. Mice received an intraperitoneal injection of BrdU (BD Pharmigen^TM^; 5 mg/kg/body weight) 24 hours before sacrifice.

**In vivo xenograft model**

Athymic female CD-1 nude mice received via subcutaneous injection 3x10^6^ of scramble or overexpressing GFP-RKO cells. After injection, the tumor formation was monitored daily over 23 days and measured by the use of a vernier caliper. After this time, mice were sacrificed, and the tumors enzymatically digested in RPMI medium supplemented with Collagenase/Dyspase (1mg/ml) (Roche#1109711300; Roche Diagnostics GmbH) and DNAase I (20μg/ml) (Roche#10104159001); Roche Diagnostics GmbH) at 37°C for 30 minutes.

**Organoid culture**

The culture of small intestinal organoids was performed as previously described^2^. The intestine was opened lengthwise, cut into 2 cm pieces, and washed in ice-cold PBS. The tissue was further cut in smaller pieces and incubated in PBS 2mM EDTA at 37°C for 30 minutes. Then the crypts were collected by shaking the tissue and filtering the solution through a 70-µm filter to remove the villus fraction. The crypts isolated were counted and plated within the Matrigel®, cultured in complete DMEM/F12 medium (B27 supplement, N2 supplement, PenStrep, HEPES, Glutamine, 500 ng/ml R-spondin1 (Peprotec), 100 µg/ml Noggin (Peprotec) and 50 ng/ml murine EGF (Peprotec)) and the budding neurospheres completely developed after seven days.

**Immunoblotting analysis**

Proteins were extracted from human and murine samples by mechanical homogenization in the Lysis Buffer (Tris HCl ph 7.4 50 Mm, EDTA ph 8 1 mM, NaCl 150 mM, 1% Triton, 0.5% Sodium Deoxycholate, 0.1% Sodium Dodecyl Sulfate) through the bead mill Tissue Lyzer II (QIAGEN). Human epithelial cells have been homogenized by disrupting the pellet mechanically by an 18G-needle syringe in the Lysis Buffer. The protein was quantified by using DC protein assay kit (Biorad) and separated on 10% SDS-polyacrylamide gel. The following primary antibodies were used: anti-mouse/human S1PR2 rabbit (1:200 in TBS-T 1X 5% milk, Acris AP01198PU-N), anti Phospho-AKT1 (Ser473) Clone (D7F10) (1:1000 in TBTS-T 3% BSA, MAB-94125 Immunological Science), PTEN (1:1000 in 3% nonfat dry milk in TBTS-T, Immunological Sciences). The levels of proteins were normalized on total AKT (1:1000 in TBS-T 1X 3% BSA; 9272 Cell Signaling Technology) and anti-Actin C-11 (1:1000 in TBS-T 1X 5% milk, Santa Cruz sc-1615) expression. Finally, the immunoreactivity was detected by an enhanced chemiluminescence reaction (ECL - Millipore) and developed by the ChemiDoc Imaging System.

**Overexpression constructs**

For the transfection of the RKO cell line, GFP-tagged lentiviral vectors (cat. no RG-2101663) harboring human sphingosine-1 phosphate receptor 2 and empty GFP-tagged as control vectors, both obtained from OriGene, were used. The production of the lentiviral particle was performed by transient transfection of 293T cells following standard protocol^4^. Briefly, 293T cells at 70% confluency were co-trasfected with 11.7 μg of the transfer plasmid, 3.5 μg of the packaging plasmid, and the same amount of envelope plasmid and 5 μg of rev-expressing plasmid by Lipofectamine. After 24 hours, the recombinant lentiviral vectors were collected, filtered, and used as a medium for the infection of the RKO cell line. The transfected cells were selected via flow cytometry sorting GFP positive cells, and infection efficiency were checked by RT-PCR. The transfected cells were used within 1-2 passages.

**RNA extraction and quantitative RT-PCR analysis**

Total RNA was extracted from human and murine samples using the RNeasy® Mini kit (Quiagen) according to the manufacturer’s protocol. The mRNA has been retrotranscribed in cDNA by using the High Capacity cDNA Reverse Transcription Kits (Applied Biosystem) and quantitative real-time PCR has been performed using the Fast SYBR® Green Master Mix (Applied Biosystems) and detected with 7900HT Sequence Detection System (Applied Biosystems). The primer sequences used are reported in the **supplementary** **Table 2**. Glyceraldehyde 3-phosphate dehydrogenase (GAPDH) gene has been used as housekeeping. The relative mRNA expression was determined by the 2^-ΔCt^ method.

**Supplementary Table 2. List of primers**

| **Gene** | **Primer sequences** |
| --- | --- |
| *hS1PR1* | Forward: 5’- ATGGTGTCCACTAGCATCCC-3’  Reverse: 5’-CGATGTTCAACTTGCCTGTGTAG-3’ |
| *hS1PR2* | Forward: 5’- ATGGGCGGCTTATACTCAGAG -3’  Reverse: 5’- GCGCAGCACAAGATGATGAT -3’ |
| *hS1PR3* | Forward: 5’- CCATTGCCATTGAGCGGACAC -3’  Reverse: 5’- TTAGCCAGCACATCCCAATCA -3’ |
| *hMMP2* | Forward: 5’- GATACCCCTTTGACGGTAAGGA-3’  Reverse: 5’- CCTTCTCCCAAGGTCCATAGC-3’ |
| *hMMP1* | Forward 5’-AGCTAGCTCAGGATGACATTGATG-3′  Reverse: 5′-GCCGATGGGCTGGACAG-3 |
| *hSOX9* | Forward: 5’-GAGGAAGTCGGTGAAGAACG-3’  Reverse: 5’-ATCGAAGGTCTCGATGTTGG-3’ |
| *hAXIN2* | Forward: 5’-CAACACCAGGCGGAACGAA -3’  Reverse: 5’-GCCCAATAAGGAGTGTAAGGACT -3’ |
| *hGAPDH* | Forward: 5’- TGTGTCCGTCGTGGATCTGA -3’  Reverse: 5’- CCTGCTTCACCACCTTCTTGA -3’ |
| *hPTEN* | Forward: 5’-GAGCGTGCAGATAATGACAAG-3’  Reverse: 5’-GATTTGACGGCTCCTCTAACTG-3’ |
| *mOLFM4* | Forward: 5’-GGAGCGCTTAGAGTACACAG-3’  Reverse: 5’- GGAGCCTCTTCTCATACACAC-3’ |
| *mLGR5* | Forward: 5’- ATGAACAACATCAGTCAGCTAC-3’  Reverse: 5’- CTCCCTTGGGAATGTGTGTC-3’ |
| *mGAPDH* | Forward: 5’- CCATGTTCGTCATGGGTGTG -3’  Reverse: 5’- CAGGGGTGCTAAGCAGTTGG -3’ |

H=human; m=murine

**Measurement of S1P**

Approximately 20–60 mg of the tumor and normal mucosa sample was homogenized using a lysis buffer containing the internal standard solution (10.0 ng/mL, sphingosine17:1, and S1P 17:1) and analyzed by LC-MS/MS. The ratio of the peak area of the analyte to the internal standard was used to quantify the calibration curves. The results are reported as pmol/mg.

as a tumor suppressor. Its loss may represent a risk factor for the development of colorectal cancer.

**Enzyme-Linked Immunosorbent Assay**

IL-6, IL17A and IFNy concentration was evaluated in serum of mice by using an enzyme-linked immunosorbent assay kit for IL-6 and IFNy detection (R&D Systems, Minneapolis, MN, USA), for IL17A (eBioscience, San Diego, CA, USA), following manufacturer instructions.

**SUPPLEMENTARY REFERENCES**

1. Klein M, Picard E, Vignaud JM, et al. Vascular endothelial growth factor gene and protein: strong expression in thyroiditis and thyroid carcinoma. J Endocrinol 1999;161:41-9.

2. Mahe MM, Aihara E, Schumacher MA, et al. Establishment of Gastrointestinal Epithelial Organoids. Curr Protoc Mouse Biol 2013;3:217-40.

3. Vetrano S, Rescigno M, Cera MR, et al. Unique role of junctional adhesion molecule-a in maintaining mucosal homeostasis in inflammatory bowel disease. Gastroenterology 2008;135:173-84.

4. Dull T, Zufferey R, Kelly M, et al. A third-generation lentivirus vector with a conditional packaging system. J Virol 1998;72:8463-71.
